# Arteriovenous fistula for haemodialysis as a predictor of *de novo* heart failure in kidney transplant recipients

**DOI:** 10.1093/ckj/sfae105

**Published:** 2024-04-18

**Authors:** Sokratis Stoumpos, Peter Van Rhijn, Kenneth Mangion, Peter C Thomson, Patrick B Mark

**Affiliations:** Renal and Transplant Unit, Queen Elizabeth University Hospital, Glasgow, UK; Renal and Transplant Unit, Queen Elizabeth University Hospital, Glasgow, UK; School of Cardiovascular and Metabolic Health, University of Glasgow, Glasgow, UK; Renal and Transplant Unit, Queen Elizabeth University Hospital, Glasgow, UK; Renal and Transplant Unit, Queen Elizabeth University Hospital, Glasgow, UK; School of Cardiovascular and Metabolic Health, University of Glasgow, Glasgow, UK

**Keywords:** arteriovenous fistula, cardiovascular, heart failure, kidney transplant

## Abstract

**Background:**

The haemodynamic effects of a functioning haemodialysis arteriovenous fistula (AVF) can cause or exacerbate heart failure (HF). We investigated whether the presence of an AVF at the time of kidney transplant (KT) is associated with *de novo* HF.

**Methods:**

This was an observational cohort study including adult patients who received a KT in the West of Scotland between 2010 and 2020. We evaluated the risk and associations of pretransplant factors with *de novo* HF, alone and as a composite cardiovascular (CV) outcome (including non-fatal myocardial infarction, non-fatal stroke, *de novo* HF and CV death). Multivariable proportional hazards regression and sensitivity analyses were used to identify independent correlates of the outcomes.

**Results:**

Among 1330 included patients, the incident rate of *de novo* HF after transplantation was 58/1000 person-years [95% confidence interval (CI) 50–67] in AVF patients (*n* = 716) compared with 33/1000 person-years (95% CI 27–41) in non-AVF patients (*n* = 614). *De novo* HF was associated with the presence of an AVF [adjusted hazard ratio (aHR) 2.14 (95% CI 1.40–3.26)], duration of dialysis [aHR 1.03/year increase (95% CI 1.01–1.04)], age at transplant [aHR 1.03/year increase (95% CI 1.02–1.05)], female sex [aHR 1.93 (95% CI 1.40–2.65)] and pretransplant diabetes [aHR 2.43 (95% CI 1.48–4.01)]. The presence of an AVF was also associated with the composite CV outcome [aHR 1.91 (95% CI 1.31–2.78)].

**Conclusions:**

The presence of an AVF may be an underrecognized modifiable predictor of *de novo* HF posttransplantation.

KEY LEARNING POINTS **What was known:**

- Data on posttransplantation *de novo* heart failure (HF) are sparse and most commonly identified in US registry data.
- Traditional risk factors associated with *de novo* HF are similar to those of the general population, but non-traditional recipient or donor-related factors are also implicated.
- *De novo* HF is associated with lower patient and kidney transplant survival.

**This study adds:**

- Using robust echocardiographic criteria, we included patients with HF with reduced and preserved ejection fraction.
- Receiving dialysis via an AVF before transplantation, among other risk factors, was associated with *de novo* HF and adverse cardiovascular outcomes posttransplantation.

**Potential impact:**

- This study suggests that the presence of a functioning AVF may have deleterious cardiac effects posttransplantation.
- Although a causal relationship was not established in this study, the risk of *de novo* HF in the presence of an AVF should be carefully evaluated as part of routine pretransplant assessment.

## INTRODUCTION

Although kidney transplantation considerably improves survival and quality of life when compared with chronic dialysis treatment [1], cardiovascular disease (CVD) remains the major cause of death among kidney transplant (KT) recipients [2]. Heart failure (HF) whether pre-existing or newly diagnosed posttransplant, is associated with increased mortality after transplantation [3, 4]. Studies have shown that the prevalence of pretransplant HF with reduced ejection fraction (HFrEF) in patients referred or wait-listed for transplantation may be as high as 25% [3, 5, 6]. Data on the prevalence of *de novo* HF posttransplant are outdated and limited due to universal lack of echocardiographic data in reported studies, which use clinical criteria or coded diagnoses for HF [4, 7, 8]. Notably, more recent studies have observed a reduction in *de novo* HF posttransplantation over time, most likely as a result of improvements in pretransplantation cardiac risk stratification and modification [9, 10], dialysis care [11] and contemporaneous improvements in posttransplantation kidney care [8, 12, 13]. This occurs despite the aging and increased comorbidity burdens among KT recipients [14].

Traditional risk factors associated with *de novo* HF posttransplant include age, female, African American, smoking, obesity, atherosclerotic disease, diabetes and hypertension [4, 7, 15]. Non-traditional risk factors include delayed graft function, suboptimal KT function and donor-related factors [4, 7, 15]. The haemodynamic effects of a functioning haemodialysis (HD) arteriovenous fistula (AVF) can cause or exacerbate HF in patients with end-stage kidney disease (ESKD) [16–18] and studies advocate AVF ligation posttransplantation to mitigate these effects [19–22]. The effect of a functioning AVF pretransplantation in the development of *de novo* HF posttransplantation has not been investigated.

The goal of this study was to evaluate the risk of *de novo* HF posttransplantation using echocardiographic data and determine pretransplant demographic and clinical factors predictive of *de novo* HF. This study sought to specifically determine whether receiving dialysis via an AVF before transplantation is associated with new-onset HF posttransplantation.

## MATERIALS AND METHODS

### Data sources

Data were obtained from the West of Scotland Electronic Renal Patient Record (SERPR) to identify all KT recipients and linked with the Scottish Electronic Health Records using the Community Health Index number. The West of Scotland KT program is a regional service including five health boards (NHS Greater Glasgow & Clyde, Forth Valley, Dumfries & Galloway, Lanarkshire, Ayrshire & Arran) and occasionally other parts of Scotland covering a population of 1.7 million. Since the year 2000, >3000 KTs were performed in the Glasgow Transplant Unit and 150–200 patients are transplanted each year.

This study is reported according to the Strengthening the Reporting of Observational Studies in Epidemiology guidelines (see Supplementary data).

### Study population

We included adult (≥18 years of age) recipients of a KT who underwent transplantation from 1 January 2010 to 31 March 2020. During this era, all patients were treated with the SYMPHONY immunosuppressive regime of induction with an anti-interleukin-2 receptor monoclonal antibody (or anti-thymocyte globulin in case of high immunological risk), an antiproliferative, tacrolimus and prednisolone, except where clinical circumstances dictated alternative therapy [23]. Patients with pre-existing HF (before undergoing kidney transplantation) were excluded. We also excluded patients with missing data on the dialysis modality and type of vascular access used prior to transplantation and those receiving dialysis via a synthetic graft. We ended observation on 1 April 2021, to allow at least 1 year for detection of the primary outcome. We restricted our study in the pre-coronavirus disease 2019 era to avoid the impact of the pandemic on our transplant program and healthcare services. As we analysed routine care data, institutional ethical approval was waived but the study was approved by the data protection officer on behalf of the NHS Greater Glasgow & Clyde health board (Caldicott Guardian approval reference number NHSGGC/1061/11Jun21).

### Study variables and outcome definitions

#### Baseline variables

Information on age, gender, blood pressure, body mass index (BMI), cause of ESKD, pretransplantation dialysis modality and vascular access, duration of dialysis, diabetes, ischaemic heart disease, atrial fibrillation, cerebrovascular disease, peripheral arterial disease and HF diagnoses before and after transplantation was obtained through the SERPR. The most recent blood results up to 90 days pretransplantation, medications (including immunosuppressants) up to 90 days posttransplantation, mortality and KT outcomes were also retrieved. All echocardiography and chest X-ray reports and N-terminal prohormone of brain natriuretic peptide (NT-proBNP) levels were retrieved from the electronic patient records. The last echocardiography before transplantation (performed within 2 years) and the most recent posttransplantation (performed within 5 years) were used to define HF outcomes. Structured Query Language (SQL) and visual coding strategies were used to capture relevant ‘text strings’ from echocardiography and chest X-ray reports and physician reported diagnoses.

#### Outcome definitions

Primary outcomes included *de novo* HF, alone and as a composite cardiovascular (CV) outcome. HF was defined using a combination of echocardiography criteria, as per the 2021 European Society of Cardiology (ESC) guidelines for the diagnosis and treatment of acute and chronic HF [24], physician-reported diagnoses and radiological criteria (Table 1). The same criteria were used to define HF pre- and posttransplantation. We included patients with both HFrEF and HF with preserved ejection fraction (HFpEF). To define HFpEF we used markers of structural heart disease [left ventricular hypertrophy (LVH) or left atrium (LA) enlargement] or diastolic dysfunction. We have used measures of the LA area as a marker of LA enlargement, as LA volumes were not available in our database to estimate the LA volume index (LAVI) [25]. The CV outcome included a composite of non-fatal myocardial infarction (MI), non-fatal stroke, *de novo* HF and CV death (death from MI, stroke, HF or arrhythmia). Secondary outcomes included death, KT failure and death-censored KT failure. Observations were censored at lost to follow-up, death, KT failure or end of observation.

**Table 1:** Criteria used to define HF^a^ (adapted from the 2021 ESC guidelines for the diagnosis and treatment of acute and chronic heart failure [24])

Criterion	Parameter	Threshold
Echocardiography criteria for HFrEF and HFmrEF	HFrEF (LVEF ≤40%) or HFmrEF (LVEF 41–49%)	LVEF <50% using the modified Simpson method or moderate/severe LVSD using visual estimation
Echocardiography criteria for HFpEF	HFpEF (LVEF ≥50%)	LVEF ≥50% using the modified Simpson method or normal LV function using visual estimation
	LV mass index	≥95 g/m^2^ (female), ≥115 g/m^2^ (male)
	LA area	>20 cm^2^
	E:e′ ratio	>9
	NT-proBNP	>125 pg/ml (SR) or >365 pg/ml (AF)
	TR peak velocity	>2.8 m/s
Physician reported diagnoses	Clinical syndrome of HF	Diagnoses strings: LV failure, congestive cardiac failure, heart failure, cardiac failure, LVSD, pulmonary oedema, fluid overload
Radiological criteria^b^	Objective evidence of cardiogenic, pulmonary or systemic congestion by chest X-ray	Chest X-ray strings: pulmonary oedema, pulmonary congestion, fluid overload, cardiac decompensation, cardiac failure

a After the first month of transplantation.

b Excluded chest X-ray performed during admissions with intercurrent illness or KT failure.

AF: atrial fibrillation; HFmrEF, heart failure with mildly reduced ejection fraction; LVSD: left ventricular systolic dysfunction; SR: sinus rhythm; TR: tricuspid regurgitation.

### Patient allocation

We divided the patients into two groups based on the dialysis modality and type of vascular access used prior to transplantation: patients on HD via an AVF versus all the rest [patients on HD via a central venous catheter (CVC), on peritoneal dialysis (PD), with a prior KT or low clearance]. In a subgroup analysis, we only included patients on HD (dialysis via an AVF versus CVC), as patients with pre-emptive transplantation or on PD tend to have better outcomes and lower mortality after KT. The last renal replacement therapy modality and last dialysis access entries before the date of transplantation were used to ascertain the use of an AVF (or not) immediately prior to transplantation. As a verification measure, the first dialysis access used within the first 7 days of transplantation (for patients with delayed graft function) was also retrieved.

### Statistical analyses

Continuous variables were presented as either mean with standard deviation (SD) or median with interquartile range (IQR). Categorical variables were expressed as percentages.

Due to the different durations of follow-up, incidence rates of *de novo* HF were estimated using person-time (years) of the population at risk.

Values were missing in <20 individuals, except for primary renal disease [*n* = 164 (12%)] and BMI [*n* = 285 (21%)]. Missing variables were handled through multiple imputations [chained equations (*n* = 20)]. The imputation model included all covariates, the outcome and the time of follow-up.

We plotted survival curves using Kaplan–Meier estimators and applied proportional hazards (Cox) regression models to assess the effect of a functioning AVF at the time of transplantation compared with no AVF on the risk for *de novo* HF, alone and as a composite CV outcome. In a sensitivity analysis, we used Fine–Gray models [26] to assess the effect of AVFs on *de novo* HF and composite CV outcome, accounting for competing risks for KT failure and death. The same analyses were used for secondary outcomes (death, KT failure and death-censored KT failure).

To assess whether the observed effects are consistent across the different patient subgroups, we performed sensitivity analyses for different types of HF (HFpEF versus HFrEF), in HD patients dialysing via an AVF versus those dialysing via a CVC (*n* = 923) and by AVF type [radiocephalic, brachiocephalic, brachiobasilic, other (*n* = 716)].

Receiver operating characteristics (ROC) curves were generated to determine the predictive value of AVF, age and duration of dialysis in *de novo* HF.

For all analyses, *P*-values of .05 were considered statistically significant. All analyses were performed using Stata 16.1 (StataCorp, College Station, TX, USA).

## RESULTS

### Baseline patient characteristics

**Figure 1:**
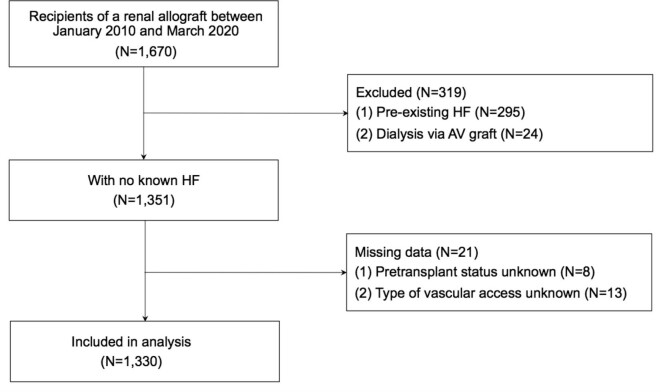
Creation of the study cohort.

We identified 1670 patients who underwent kidney transplantation during the study period. From this cohort, we excluded 295 patients with pretransplantation HF and 24 patients who were receiving HD via an AV graft. Another 21 patients were excluded due to missing data (Fig. 1). The remaining 1330 patients served as the study cohort. The demographic and clinical characteristics of study participants by AVF status at the time of transplantation are summarized in Table 2. Males were more likely to have an AVF compared with females and patients with an AVF had been receiving dialysis for longer. In the non-AVF group, 220 (35.8%) patients received a pre-emptive KT. The median time between the last echocardiography and transplantation was 13.6 months (IQR 6.3–23) and the median time between transplantation and the most recent echocardiography was 39.8 months (IQR 15.6–60). The main echocardiographic characteristics and NT-proBNP levels before and after transplantation are shown in Table 3. Regression of LVH and a reduction in LV volumes were observed post-transplantation despite no differences in left ventricular ejection fraction (LVEF), and these changes were more pronounced in the no-AVF group. Furthermore, subtle changes in measures of elevated left ventricular filling pressures were noted post-transplantation in both groups [elevated LA area, E:e′ (early filling velocity on transmitral Doppler/early relaxation velocity on tissue Doppler) ratio and TR peak velocity].

**Table 2:** Patient characteristics by AVF status at the time of KT

Characteristics	AVF (*n* = 716)	No AVF (*n* = 614)	*P*-value
Demographics			
Age (years), median (IQR)	52.5 (42.5–60.7)	51.5 (40.0–59.3)	.02
Male, *n* (%)	461 (64.4)	332 (54.1)	<.001
Systolic BP (mmHg), mean (SD)	142 (27)	145 (29)	.04
Diastolic BP (mmHg), mean (SD)	77 (16)	82 (16)	<.001
BMI (kg/m^2^), median (IQR)	25.9 (22.7–30.2)	26.6 (23.2–30.5)	.11
Primary renal disease, *n* (%)			
Glomerulonephritis	255 (35.6)	194 (31.6)	.47
Congenital	89 (12.4)	91 (14.8)	
Polycystic kidney disease	106 (14.8)	106 (17.3)	
Diabetic nephropathy	78 (10.9)	74 (12.1)	
Renovascular disease	35 (4.9)	24 (3.9)	
Unknown	105 (14.7)	88 (14.3)	
Other	48 (6.7)	37 (6.0)	
Comorbid conditions, pretransplantation, *n* (%)			
Diabetes	96 (13.4)	91 (14.8)	.46
Myocardial infarction	58 (8.1)	37 (6.0)	.14
Atrial fibrillation	72 (10.1)	49 (8.0)	.005
Stroke	30 (4.2)	22 (3.6)	.57
Peripheral arterial disease	20 (2.8)	11 (1.8)	.23
Pretransplant status (for non-AVF group), *n* (%)			
Predialysis		220 (35.8)	
HD via CVC		207 (33.7)	
PD		172 (28.0)	
Prior KT		15 (2.5)	
Renal replacement therapy duration (months), median (IQR)^a^	37 (19–75)	26 (11–61)	<.001
Medications, pretransplantation, *n* (%)			
ACEi/ARB	97 (13.5)	112 (18.2)	.02
Loop diuretic	56 (7.8)	103 (16.8)	<.001
Calcium channel blocker	170 (23.7)	214 (34.9)	<.001
Beta-blocker	200 (27.9)	217 (35.3)	.004
Statin	285 (39.8)	243 (39.6)	.93
Maintenance immunosuppression, *n* (%)			
Tacrolimus–MMF–prednisolone	465 (64.9)	356 (58.0)	.009
Tacrolimus–prednisolone	149 (20.8)	151 (24.6)	.1
Tacrolimus–MMF	32 (4.5)	29 (4.7)	.83
Other regimens	70 (9.8)	78 (12.7)	.52
Blood tests, pretransplantation, median (IQR)^b^			
Haemoglobin (g/l)	116 (104–126)	110 (99–120)	<.001
Ferritin (μg/l)	354 (203–614)	242 (127–414)	<.001
Albumin (g/l)	37 (34–40)	36 (33–39)	.003
CRP (mg/l)	6.0 (2.2–11.0)	4.0 (2.0–10.0)	.001
Total cholesterol (mmol/l)^c^	4.0 (3.4–4.7)	4.3 (3.5–5.2)	<.001
Adjusted calcium (mmol/l)	2.3 (2.2–2.4)	2.4 (2.3–2.5)	<.001
Phosphate (mmol/l)	1.4 (1.1–1.8)	1.6 (1.3–1.9)	<.001

a Excludes pre-dialysis patients (*n* = 220).

b Up to 90 days pretransplantation.

c Up to 1 year pretransplantation.

ACEi: angiotensin-converting enzyme inhibitor; ARB: angiotensin receptor blocker; BP: blood pressure; MMF: mycophenolate mofetil.

**Table 3:** Echocardiographic characteristics and NT-proBNP levels before and after kidney transplantation

	Pretransplant			Posttransplant		
Characteristics	All	No AVF	AVF	All	No AVF	AVF
*n*	496	244	252	532	208	324
LVEF (%)	59.6 (8.4)	60.6 (7.0)	58.6 (9.5)	58.8 (10.6)	58.8 (12.0)	58.7 (9.8)
LV mass (g)	168.1 (36.1)	166.6 (33.0)	169.6 (38.8)	163.4 (39.1)	157.9 (38.0)	166.8 (39.5)
LV mass index (g/m^2^)	91.1 (22.5)	90.9 (20.7)	91.4 (24.1)	88.3 (23.8)	85.4 (22.8)	90.2 (24.2)
LV end-diastolic diameter (cm)	4.8 (0.7)	4.7 (0.6)	4.9 (0.7)	4.7 (0.7)	4.6 (0.7)	4.8 (0.7)
LV end-diastolic volume (ml)	110.5 (38.4)	109.3 (32.0)	111.6 (43.4)	100.8 (32.9)	91.6 (33.3)	107.1 (31.3)
LV end-systolic diameter (cm)	3.2 (0.6)	3.1 (0.6)	3.3 (0.6)	3.1 (0.7)	3.0 (0.7)	3.1 (0.7)
LV end-systolic volume (ml)	42.3 (24.5)	39.8 (19.6)	44.4 (28.0)	38.1 (21.3)	33.5 (25.1)	40.4 (18.9)
LA area (cm^2^)	20.1 (5.6)	19.0 (5.1)	21.2 (5.9)	22.1 (6.7)	21.0 (6.0)	22.7 (7.0)
E:e′ ratio	9.7 (4.1)	9.3 (3.6)	10.1 (4.4)	11.9 (5.9)	11.3 (5.1)	12.3 (6.2)
E:A ratio	1.1 (0.4)	1.1 (0.4)	1.1 (0.4)	1.0 (0.4)	1.0 (0.5)	1.0 (0.3)
TR peak velocity (m/s)	2.4 (0.5)	2.4 (0.5)	2.4 (0.5)	2.6 (0.6)	2.6 (0.5)	2.6 (0.6)
NT-proBNP (pg/ml), median (IQR)	191 (118–345)	187 (123–300)	235 (118–445)	289 (167–755)	267 (187–768)	334 (130–720)

Values are presented as mean (SD) unless stated otherwise.

E:A ratio: early (E):late (A) ventricular filling velocities; TR: tricuspid regurgitation.

Compared with patients with HFrEF, those with HFpEF were older (median age 58 versus 56 years), predominantly females (53% versus 45%), with higher LVEF (mean 63% versus 45%) and lower NT-proBNP levels (median 340 versus 2674 pg/ml).

### Incidence of *de novo* HF after transplantation

Over a median follow-up of 4.2 years, *de novo* HF occurred in 175/716 (24.4%) patients with an AVF at the time of transplantation compared with 91/614 (14.8%; *P* < .001) patients with no AVF. Similarly, the composite CV outcome occurred in 201/716 (28.1%) patients with an AVF compared with 107/614 (17.4%; *P* < .001) patients with no AVF. Secondary outcomes were more frequent in the AVF group (Table 4).

**Table 4:** Patient outcomes by AVF status at time of KT

Outcomes	AVF (*n* = 716)	No AVF (*n* = 614)	*P*-value
Primary, *n* (%)			
De novo heart failure	175 (24.4)	91 (14.8)	<.001
Composite CV outcome	201 (28.1)	107 (17.4)	<.001
Secondary, *n* (%)			
Death	124 (17.3)	74 (12.1)	.007
KT failure	197 (27.5)	124 (20.2)	.002
Death-censored KT failure	95 (13.3)	68 (11.1)	.22

The incident rate of *de novo* HF after transplantation was 46/1000 person-years (58/1000 person-years in patients with an AVF versus 33/1000 person-years in patients with no AVF; Table 5). For the composite CV outcome, the incident rate was 55/1000 person-years (68/1000 person-years in patients with an AVF versus 40/1000 person-years in patients with no AVF; Supplementary Table S1).

**Table 5:** Factors associated with *de novo* HF after KT following risk adjustment

Factors	Incidence rates, person-years (95% CI)	Univariable HR (95% CI)	Multivariable HR (95% CI)	Competing risks HR (95% CI)
AVF				
No	32.9 (26.8–40.5)	Reference		
Yes	57.9 (50.0–67.2)	1.76 (1.37–2.27), *P* < .001	2.14 (1.40–3.26) *P* < .001	1.91 (1.28–2.86), *P* = .002
Duration of dialysis, per year increase		1.03 (1.01–1.04), *P* < .001	1.03 (1.01–1.04), *P* < .001	1.02 (1.01–1.04), *P* < .001
Sex				
Male	39.7 (33.6–46.8)	Reference		
Female	55.9 (47.0–66.6)	1.41 (1.11–1.79), *P* = .005	1.93 (1.40–2.65), *P* < 0.001	1.76 (1.29–2.41), *P* < .001
Age, per year increase		1.04 (1.03–1.05), *P* < .001	1.03 (1.02–1.05), *P* < .001	1.03 (1.01–1.04), *P* < .001
Renal diagnosis, category				
GN	42. 4 (34.2–52.5)	Reference		
Congenital	43.2 (34.6–54.0)	1.01 (0.74–1.37), *P* = .96	0.87 (0.59–1.28), *P* = .48	0.91 (0.61–1.33), *P* = .62
Vascular	55.9 (41.6–75.1)	1.30 (0.90–1.88), *P* = .16	0.60 (0.34–1.05), *P* = .07	0.60 (0.33–1.08), *P* = .09
Other	49.5 (38.5–63.6)	1.13 (0.81–1.58), *P* = .46	1.02 (0.68–1.51), *P* = .93	0.97 (0.66–1.45), *P* = .89
Diabetes				
No	42.3 (37.1–48.4)	Reference		
Yes	74.6 (56.4–98.7)	1.81 (1.32–2.47), *P* < .001	2.43 (1.48–4.01), *P* < .001	2.11 (1.23–3.62), *P *= .007
Myocardial infarction				
No	42.9 (37.8–48.8)	Reference		
Yes	89.6 (64.0–125.4)	2.04 (1.42–2.92), *P* < .001	1.33 (0.85–2.08), *P* = .21	1.44 (0.92–2.23), *P* = .11
Atrial fibrillation				
No	44.2 (39.0–50.1)	Reference		
Yes	105.2 (66.3–166.9)	2.35 (1.46–3.79), *P* < .001	1.36 (0.75–2.47), *P* = 0.31	1.39 (0.77–2.50), *P* = .27
Stroke				
No	44.6 (39.4–50.5)	Reference		
Yes	85.7 (53.3–137.9)	1.92 (1.17–3.14), *P* = .009	0.57 (0.26–1.25), *P* = .16	0.58 (0.28–1.22), *P* = .15
Peripheral arterial disease				
No	45.9 (40.6–51.8)	Reference		
Yes	52.1 (23.4–116.0)	1.17 (0.52–2.62), *P* = .71	0.70 (0.27–1.81), *P* = .47	0.69 (0.26–1.85), *P* = .46
Systolic blood pressure, per mmHg increase		1.00 (0.99–1.01), *P* = .48	1.00 (0.99–1.01), *P* = .31	1.00 (1.00–1.01), *P* = .27
Diastolic blood pressure, per mmHg increase		0.99 (0.98–0.99), *P* = .03	0.99 (0.98–1.01), *P* = .35	1.00 (0.98–1.01), *P* = .42
Haemoglobin, per g/L increase		1.00 (0.99–1.00), *P* = .94	1.00 (0.99–1.00), *P* = .54	1.00 (0.99–1.00), *P* = .97
Albumin, per g/L increase		0.97 (0.95–0.99), *P* = .02	0.99 (0.96–1.03), *P* = .71	1.01 (0.97–1.04), *P* = .78
ACEi/ARB				
No	46.0 (40.5–52.4)	Reference		
Yes	45.9 (32.9–63.9)	0.99 (0.69–1.42), *P* = .97	1.41 (0.89–2.25), *P* = .15	1.35 (0.85–2.15), *P* = .21
Triple (standard) immunosuppression regimen				
No	47.6 (39.4–57.6)	Reference		
Yes	45.0 (38.5–52.5)	0.98 (0.77–1.26), *P* = .89	0.93 (0.69–1.26), *P* = .65	1.03 (0.76–1.38), *P* = .87

ACEi: angiotensin-converting enzyme inhibitor; ARB: angiotensin receptor blocker; GN: glomerulonephritis.

**Figure 2:**
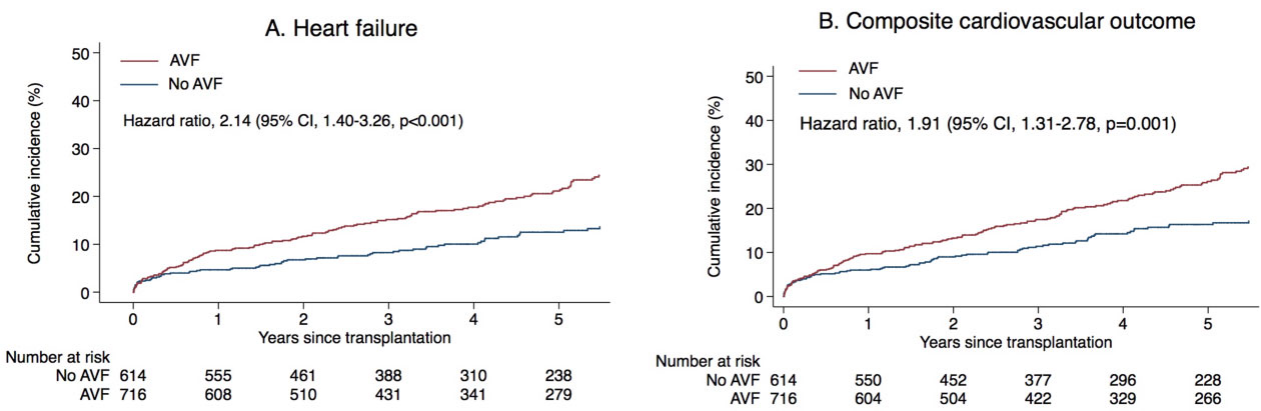
Survival curves were plotted using Kaplan–Meier estimators. HRs and 95% CIs were estimated using Cox proportional hazards regression models. The models were adjusted for baseline factors at the time of transplant. **(A)** HF. **(B)** Composite cardiovascular outcome (non-fatal MI, non-fatal stroke, *de novo* HF and CV death).

The cumulative incidences of *de novo* HF and the composite CV outcome using Kaplan–Meier failure estimates are shown in Fig. 2.

### Independent predictors for *de novo* HF

Multivariable analysis demonstrated five factors predicting *de novo* HF: the presence of an AVF, the duration of dialysis, increasing age, female sex and pretransplant diabetes. When adjusted for demographic data, duration of dialysis, primary renal diagnosis, comorbid conditions, haemoglobin and albumin levels and medications (angiotensin-converting enzyme inhibitors or angiotensin receptor blockers and immunosuppressants), the risk for developing *de novo* HF was higher for patients with an AVF at the time of transplantation compared with those with no AVF {adjusted hazard ratio [aHR] 2.14 [95% confidence interval (CI) 1.40–3.26]} when death and KT failure were treated as censoring events (Table 5). In sensitivity analysis that treated death and KT failure as competing events, the risk for *de novo* HF was also higher in patients with an AVF [aHR 1.91 (95% CI 1.28–2.86)] (Table 5). The presence of an AVF was also predictive of the composite CV outcome following adjustment of covariates [aHR 1.91 (95% CI 1.31–2.78)] (Supplementary Table S1).

On multivariable analysis, the presence of an AVF was not associated with death, KT failure or death-censored KT failure [aHR 1.28 (95% CI 0.84–1.95), aHR 1.35 (95% CI 0.96–1.90), aHR 1.22 (95% CI 0.76–1.95), respectively] (Supplementary data, Tables S2–4).

### Sensitivity analyses

The presence of an AVF was independently associated with both *de novo* HFpEF [*n* =171; aHR 2.13 (95% CI 1.34–3.38)] and HFrEF [*n* = 95; aHR 2.21 (95% CI 1.10–4.46)] (Supplementary data, Tables S5 and S6). Subgroup analysis in HD patients (*n* = 923) showed a higher adjusted risk of *de novo* HF in patients dialysing with an AVF at the time of transplantation compared with those dialysing via CVC [aHR 2.13 (95% CI 1.40–3.25)] (Supplementary data, Table S7). Further subgroup analysis in HD patients dialysing via an AVF (*n* = 716) showed no difference in the adjusted risk of *de novo* HF by AVF type (Supplementary data, Table S8).

**Figure 3:**
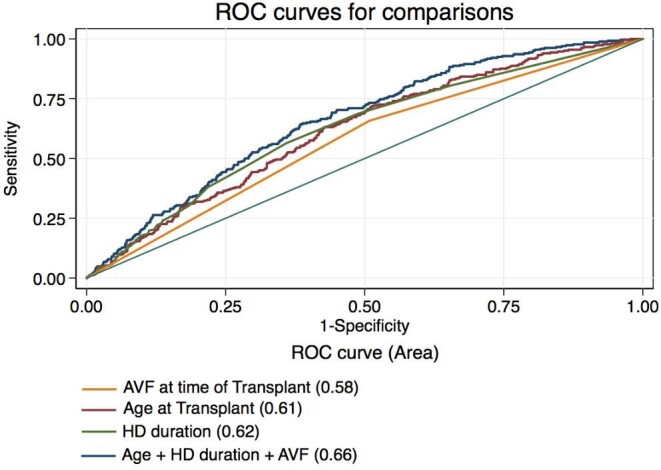
Combining age at transplant, duration of dialysis and the presence of an AVF at the time of transplantation in the model was fairly predictive of *de novo* HF (AUC 0.66). AUC: area under the curve.

ROC curves assessing the presence of an AVF pretransplant, age at transplantation and duration of dialysis showed that each of these individual factors was mildly predictive of *de novo* HF. When all three factors were considered collectively, however, the model was fairly predictive of *de novo* HF, with an area under the curve of 0.66 (95% CI 0.63–0.70) (Fig. 3).

## DISCUSSION

This study assessed the prevalence of *de novo* HF after kidney transplantation and the predictive value of a functioning AVF in *de novo* HF in a contemporary transplant population, with several noteworthy findings. First, integrating echocardiographic criteria, we reported outcomes of HF including HFpEF, which is known to be the most prevalent form of HF in CKD patients [27]. Second, using multivariable analysis, the presence of an AVF was strongly associated with *de novo* HF and adverse cardiovascular outcomes posttransplantation and the effect was independent of exposure to dialysis. Finally, in the multivariable model, age, female sex, duration of dialysis and the presence of pretransplant diabetes were also predictive of *de novo* HF. These observations have important implications and challenge current practices where AVFs are traditionally maintained in asymptomatic patients after successful kidney transplantation.

Data on the prevalence of HF before kidney transplantation are sparse and mostly derived from the US registry and single-centre studies. The reported prevalence of HF at the time of KT varies between studies and ranges from 5.8 to 11.9% [5, 6]. Lentine *et al.* [4] reported cumulative incidences of HF on the transplant waiting list of 6.5%, 12% and 32% at 6, 12 and 36 months, respectively. In another study, the prevalence of physician-reported HF in patients on the transplant waiting list was 46% at 36 months [28]. In a study using single-photon emission computed tomography (SPECT) myocardial perfusion scans for pretransplant assessment, 18% of patients had LV systolic dysfunction (defined as an LVEF ≤45%) [3]. In studies reporting long-term outcomes, HF at the time of transplantation was independently associated with a higher risk of death, cardiovascular events and KT failure [3, 4, 6].

In patients with a KT, HF has been most commonly defined in administrative and clinical databases and there are no studies using diagnostic testing, such as echocardiography. In a study including KT recipients between 1995 and 2001, Lentine *et al.* [4] reported cumulative incidences of *de novo* HF of 10.2% and 18.3% at 12 and 36 months, respectively, which is slightly higher than reported in our study (6.8% and 12.0%, respectively). This may be explained by potential misclassification of HF diagnoses when using registry International Classification of Diseases, Ninth Revision–based claims or it may represent a true decline in HF as demonstrated in a recent study, where the adjusted incidence of *de novo* HF after KT significantly declined between 1998 and 2010 [8]. Rigatto *et al.* [7] reported a cumulative incidence of *de novo* HF of 3.6% at 5 years (versus 17.2% in our study), but they only included patients who were alive and free of cardiac disease at 1-year posttransplant, thereby eliminating the highest risk interval. Studies investigating the risk for HF in specific populations have shown an increased age-adjusted risk for HF hospitalizations in African American compared with Caucasian KT recipients [15]. In a study investigating the cardiac implications of obesity in KT recipients, the 5-year cumulative incidence of HF increased significantly from 3.6% to 18.4% from the first to fourth BMI quartile [28].

Traditional risk factors associated with HF after transplantation [4, 7, 15] are also present in the general population with HF and share common pathophysiologic mechanisms [29, 30, 31]. In our study, older age (>50 years), female sex and the presence of diabetes were independently associated with *do novo* HF posttransplant. Non-traditional risk factors pertinent to the transplant population are related to chronic kidney disease (CKD), immunosuppressive agents and donor-related factors and include increased duration of dialysis before transplant, deceased donor kidney, increased donor age, delayed graft function, allograft rejection and KT failure [4, 7, 15]. This is the first study to report that the presence of a functioning AVF at the time of transplantation is a predictor of *de novo* HF, showing that the haemodynamic demands of an HD AVF are long-lasting and may precipitate HF even when not used for dialysis. A striking finding in our adjusted analysis was that the effect of an AVF was independent of dialysis duration, which was also a predictor of *de novo* HF. The presence of an AVF was also independently associated with a composite of non-fatal MI, non-fatal stroke, *de novo* HF and CV death.

We observed a higher incidence of *de novo* HF in patients receiving dialysis via an AVF compared with a CVC. This is suggestive of a deleterious effect of an AVF on the cardiovascular system rather than HD per se. Although this is an important finding, we should emphasize that patients receiving dialysis via an AVF are inherently different from those dialysing via a CVC. There are often clinical reasons why patients are more likely to have a CVC than an AVF, including old age and frailty, but also younger patients with a live donor who are expected to receive dialysis for a short period. One small cohort study suggested that patients with an AV access had an increased risk of HF compared with a CVC, even allowing for CVC patients been older [32], but this is not necessarily a consistent finding [33]. Although patients with a fistula blood flow >2 l/min or an upper arm AVF are traditionally at increased risk for the development of HF [34], in our study an AVF configuration was not associated with the risk of *de novo* of HF. However, data on fistula flows were not available for comparisons.

Studies have reported the reversal of LV remodelling and clinical cardiac dysfunction after kidney transplantation [35–39] driven by correction of the uraemic state and abolishment of the adverse effects of prolonged dialysis on myocardial function. KT recipients have a lower CV death risk and survive longer than patients on the transplant waiting list [40, 41], suggesting that the progression of CVD can be ameliorated by restoring kidney function with a transplant. In contrast, persistence of a functioning AVF after kidney transplantation has been associated with increased LV mass and LV dimensions [42], while closure of the fistula results in significant reductions in LV mass, LV end-diastolic diameter and atrial sizes [19–21].

This study is limited by its retrospective design, and as is the case with all observational studies, residual confounding is a possibility. Although we have used objective criteria of cardiac structural, functional and serological abnormalities to define HFpEF, we were unable to obtain data on LA volumes, which is a better marker of LA enlargement. Another limitation is that echocardiography was not routinely performed in all KT recipients but only in patients with symptoms suggestive of HF (by indication) and, as such, the exact time of HF diagnosis could not be accurately determined. Although AVFs were functioning at the time of transplantation, whether these fistulas were still functioning months or years posttransplant could not be verified. Despite its limitations, this study is strengthened by the use of echocardiographic data rather than clinically coded diagnoses for HF, the quality of data in our prospectively maintained database, which may approach that obtainable in a prospective cohort study, and the nearly complete follow-up. We also avoided misclassification of pre-existing HF as ‘*de novo*’ because of underascertainment of pretransplantation status, which is common in registry data. Finally we have reported on measures of LV diastolic dysfunction beyond the LVEF and, in fact, LVEF does not reflect the complex haemodynamic adaptations of the heart to the AVF [34, 43, 44].

Our study suggests that the presence of a functioning AVF at the time of transplantation may be a modifiable underrecognized risk factor for *de novo* HF after transplantation. Older age, female sex, duration of dialysis and pretransplant diabetes also predict *de novo* HF. The risk of *de novo* HF in the presence of an AVF should be carefully evaluated as part of routine pretransplant assessment. Towards this, a systematic evaluation has recently been proposed suggesting prophylactic AVF ligation starting at 12 months after transplantation if risk factors for HF are present [45]. Whether closure of an ‘unnecessary’ AVF in stable KT recipients would prevent HF could be an area for future research and must be weighed against its generalizability to diverse transplant populations, the risk of losing a dialysis access site and potentially high implementation costs.

## Supplementary Material

sfae105_Supplemental_File

## Data Availability

The data underlying this article will be shared upon reasonable request to the corresponding author.
