# Supplementary material for: Arteriovenous fistula for haemodialysis as a predictor of de novo heart failure in kidney transplant recipients
Source: Clin Kidney J. 2024 Apr 18;17(5):sfae105. doi: 10.1093/ckj/sfae105 (PMC11087827; doi:10.1093/ckj/sfae105)
Supplement: sfae105_Supplemental_File [file sfae105_supplemental_file.docx]

**Supplemental Material**

**Arteriovenous fistula for haemodialysis as a predictor of *de novo* heart failure in kidney transplant recipients**

**Table S1 …………………………………………………………………………2**

**Table S2 …………………………………………………………………………6**

**Table S3 …………………………………………………………………………9**

**Table S4 ……………………………………………………………………….12**

**Table S5 ……………………………………………………………………….15**

**Table S6 ……………………………………………………………………….19**

**Table S7 ……………………………………………………………………….23**

**Table S8 ……………………………………………………………………….26**

**STROBE statement ……………………………………………………….30**

**Table S1.** Factors associated with composite CV outcome after KT following risk-adjustment

|  | | Incidence rates, person-years (95% CI) | Univariable HR (95% CI) | Multivariable HR (95% CI) | Competing risks HR (95% CI) |
| --- | --- | --- | --- | --- | --- |
| AVF | No | 39.8 (32.9-48.1) | Reference | | |
|  | Yes | 68.1 (59.3-78.2) | 1.71 (1.35-2.16, p<0.001) | 1.91 (1.31-2.78, p=0.001) | 1.71 (1.19-2.47, p=0.004) |
| Duration of dialysis, per year increase |  |  | 1.02 (1.01-1.03, p<0.001) | 1.02 (1.01-1.04, p<0.001) | 1.02 (1.01-1.04, p<0.001) |
| Sex | Male | 48.9 (42.1-56.9) | Reference | | |
|  | Female | 63.4 (53.7-74.8) | 1.29 (1.03-1.62, p=0.02) | 1.76 (1.31-2.37, p<0.001) | 1.62 (1.21-2.16, p=0.001) |
| Age, per year increase |  |  | 1.04 (1.03-1.05, p<0.001) | 1.03 (1.02-1.05, p<0.001) | 1.03 (1.02-1.04, p<0.001) |
| Renal diagnosis, category | GN | 48.3 (39.4-59.1) | Reference | | |
|  | Congenital | 48.0 (38.8-59.5) | 0.99 (0.74-1.33, p=0.94) | 0.90 (0.62-1.30, p=0.57) | 0.92 (0.64-1.32, p=0.65) |
|  | Vascular | 72.5 (55.6-94.4) | 1.48 (1.06-2.06, p=0.02) | 0.58 (0.34-0.98, p=0.04) | 0.58 (0.33-1.01, p=0.06) |
|  | Other | 63.3 (50.5-79.3) | 1.29 (0.95-1.74, p=0.10) | 1.10 (0.77-1.59, p=0.60) | 1.04 (0.72-1.50, p=0.82) |
| Diabetes | No | 48.6 (42.9-55.1) | Reference | | |
|  | Yes | 103.0 (80.6-131.6) | 2.13 (1.62-2.82, p<0.001) | 2.81 (1.79-4.43, p<0.001) | 2.47 (1.53-3.99, p<0.001) |
| Myocardial infarction | No | 50.9 (45.1-57.3) | Reference | | |
|  | Yes | 110.5 (80.7-151.2) | 2.13 (1.52-2.99, p<0.001) | 1.31 (0.85-2.00, p=0.22) | 1.32 (0.86-2.04, p=0.21) |
| Atrial fibrillation | No | 52.4 (46.7-58.8) | Reference | | |
|  | Yes | 126.7 (82.6-194.3) | 2.37 (1.52-3.70, p<0.001) | 1.40 (0.80-2.42, p=0.24) | 1.46 (0.85-2.50, p=0.17) |
| Stroke | No | 52.8 (47.1-59.3) | Reference | | |
|  | Yes | 110.3 (70.3-172.9) | 2.05 (1.29-3.26, p=0.002) | 0.73 (0.36-1.46, p=0.37) | 0.69 (0.35-1.37, p=0.29) |
| Peripheral arterial disease | No | 53.9 (48.1-60.3) | Reference | | |
|  | Yes | 91.2 (49.1-169.5) | 1.70 (0.90-3.19, p=0.10) | 0.88 (0.39-1.98, p=0.75) | 0.88 (0.37-2.09, p=0.78) |
| Systolic blood pressure, per mmHg increase |  |  | 1.00 (0.99-1.01, p=0.50) | 1.00 (0.99-1.01, p=0.58) | 1.00 (1.00-1.01, p=0.47) |
| Diastolic blood pressure, per mmHg increase |  |  | 0.99 (0.98-1.00, p=0.09) | 1.00 (0.99-1.01, p=0.95) | 1.00 (0.99-1.01, p=0.98) |
| Haemoglobin, per g/L increase |  |  | 1.00 (0.99-1.00, p=0.51) | 1.00 (0.99-1.00, p=0.23) | 1.00 (0.99-1.00, p=0.51) |
| Albumin, per g/L increase |  |  | 0.97 (0.94-0.99, p=0.005) | 1.00 (0.97-1.03, p=0.94) | 1.01 (0.97-1.04, p=0.68) |
| ACEi/ARB | No | 55.1 (48.9-62.1) | Reference | | |
|  | Yes | 51.3 (37.3-70.5) | 0.91 (0.65-1.28, p=0.60) | 1.14 (0.72-1.81, p=0.57) | 1.08 (0.69-1.71, p=0.73) |
| Triple (standard) immunosuppression regimen | No | 54.1 (45.2-64.8) | Reference | | |
|  | Yes | 54.9 (47.6-63.3) | 1.04 (0.82-1.30, p=0.77) | 0.97 (0.73-1.28, p=0.82) | 1.07 (0.81-1.42, p=0.62) |
| ACEi, angiotensin-converting enzyme inhibitor; ARB, angiotensin receptor blocker; AVF, arteriovenous fistula; CV, cardiovascular; GN, glomerulonephritis; KT, kidney transplantation | | | | | |

**Table S2.** Factors associated with death after KT following risk-adjustment

|  | | Incidence rates, person-years (95% CI) | Univariable HR (95% CI) | Multivariable HR (95% CI) |
| --- | --- | --- | --- | --- |
| AVF | No | 23.1 (18.4-29.1) | Reference | |
|  | Yes | 32.7 (27.4-39.0) | 1.42 (1.07-1.90, p=0.02) | 1.28 (0.84-1.95, p=0.25) |
| Duration of dialysis, per year increase |  |  | 1.02 (1.00-1.03, p=0.02) | 1.01 (0.99-1.03, p=0.28) |
| Sex | Male | 27.0 (22.5-32.5) | Reference | |
|  | Female | 30.3 (24.5-37.5) | 1.12 (0.85-1.49, p=0.43) | 1.53 (1.06-2.20, p=0.02) |
| Age, per year increase |  |  | 1.05 (1.04-1.07, p<0.001) | 1.05 (1.03-1.07, p<0.001) |
| Renal diagnosis, category | GN | 21.6 (16.4-28.5) | Reference | |
|  | Congenital | 19.2 (14.2-26.0) | 0.87 (0.58-1.31, p=0.50) | 0.72 (0.43-1.20, p=0.21) |
|  | Vascular | 45.1 (33.3-61.0) | 2.13 (1.42-3.21, p<0.001) | 0.91 (0.50-1.68, p=0.77) |
|  | Other | 41.6 (32.5-53.3) | 1.86 (1.28-2.69, p=0.001) | 1.61 (1.05-2.48, p=0.03) |
| Diabetes | No | 24.1 (20.5-28.3) | Reference | |
|  | Yes | 61.3 (46.3-81.1) | 2.71 (1.95-3.75, p<0.001) | 2.39 (1.43-4.00, p=0.001) |
| Myocardial infarction | No | 25.7 (22.0-29.9) | Reference | |
|  | Yes | 64.8 (45.6-92.2) | 2.51 (1.71-3.69, p<0.001) | 1.15 (0.69-1.92, p=0.58) |
| Atrial fibrillation | No | 27.8 (24.1-32.1) | Reference | |
|  | Yes | 43.6 (23.5-81.0) | 1.58 (0.84-2.98, p=0.16) | 0.98 (0.47-2.06, p=0.96) |
| Stroke | No | 27.4 (23.7-31.7) | Reference | |
|  | Yes | 54.8 (31.8-94.5) | 2.07 (1.18-3.63, p=0.01) | 1.40 (0.71-2.75, p=0.33) |
| Peripheral arterial disease | No | 27.8 (24.1-32.0) | Reference | |
|  | Yes | 55.2 (27.6-110.4) | 2.09 (1.03-4.24, p=0.04) | 0.70 (0.27-1.82, p=0.46) |
| Systolic blood pressure, per mmHg increase |  |  | 1.00 (0.99-1.01, p=0.87) | 1.00 (0.99-1.01, p=0.94) |
| Diastolic blood pressure, per mmHg increase |  |  | 0.99 (0.98-0.99, p=0.02) | 1.00 (0.98-1.01, p=0.75) |
| Haemoglobin, per g/L increase |  |  | 1.00 (0.99-1.00, p=0.41) | 0.99 (0.98-0.99, p=0.02) |
| Albumin, per g/L increase |  |  | 0.92 (0.90-0.95, p<0.001) | 0.96 (0.92-0.99, p=0.03) |
| ACEi/ARB | No | 29.1 (25.1-33.7) | Reference | |
|  | Yes | 23.1 (15.1-35.4) | 0.79 (0.50-1.24, p=0.30) | 0.76 (0.39-1.49, p=0.42) |
| Triple (standard) immunosuppression regimen | No | 32.1 (26.1-39.4) | Reference | |
|  | Yes | 25.8 (21.3-31.1) | 0.82 (0.62-1.09, p=0.17) | 0.89 (0.63-1.26, p=0.52) |
| ACEi, angiotensin-converting enzyme inhibitor; ARB, angiotensin receptor blocker; AVF, arteriovenous fistula; GN, glomerulonephritis; KT, kidney transplantation | | | | |

**Table S3.** Factors associated with kidney transplant failure after KT following risk-adjustment

|  | | Incidence rates, person-years (95% CI) | Univariable HR (95% CI) | Multivariable HR (95% CI) |
| --- | --- | --- | --- | --- |
| AVF | No | 42.1 (35.3-50.3) | Reference | |
|  | Yes | 56.9 (49.5-65.4) | 1.36 (1.09-1.70, p=0.007) | 1.35 (0.96-1.90, p=0.08) |
| Duration of dialysis, per year increase |  |  | 1.01 (1.00-1.02, p=0.06) | 1.00 (0.99-1.02, p=0.81) |
| Sex | Male | 48.7 (42.2-56.2) | Reference | |
|  | Female | 52.2 (44.0-62.0) | 1.07 (0.86-1.33, p=0.56) | 1.27 (0.95-1.68, p=0.11) |
| Age, per year increase |  |  | 1.02 (1.01- 1.03, p<0.001) | 1.02 (1.01-1.03, p=0.004) |
| Renal diagnosis, category | GN | 42.5 (34.6-52.0) | Reference | |
|  | Congenital | 39.2 (31.4-48.9) | 0.92 (0.68-1.24, p=0.57) | 0.80 (0.55-1.17, p=0.25) |
|  | Vascular | 63.6 (48.8-82.8) | 1.48 (1.06-2.07, p=0.02) | 0.88 (0.54-1.45, p=0.63) |
|  | Other | 69.8 (57.1-85.4) | 1.62 (1.22-2.16, p=0.001) | 1.43 (1.02-2.00, p=0.04) |
| Diabetes | No | 46.1 (40.8-52.0) | Reference | |
|  | Yes | 80.6 (62.6-103.8) | 1.75 (1.32-2.33, p<0.001) | 1.69 (1.09-2.62, p=0.02) |
| Myocardial infarction | No | 48.5 (43.2-54.4) | Reference | |
|  | Yes | 71.4 (50.8-100.4) | 1.46 (1.02-2.10, p=0.04) | 0.85 (0.53-1.36, p=0.50) |
| Atrial fibrillation | No | 49.9 (44.6-55.8) | Reference | |
|  | Yes | 56.9 (32.3-100.2) | 1.13 (0.63-2.01, p=0.68) | 0.97 (0.50-1.86, p=0.93) |
| Stroke | No | 48.9 (43.7-54.8) | Reference | |
|  | Yes | 84.1 (53.0-133.5) | 1.72 (1.07-2.77, p=0.03) | 1.23 (0.70-2.17, p=0.47) |
| Peripheral arterial disease | No | 49.3 (44.1-55.1) | Reference | |
|  | Yes | 89.6 (49.6-161.8) | 1.82 (1.00-3.32, p=0.051) | 1.10 (0.52-2.35, p=0.80) |
| Systolic blood pressure, per mmHg increase |  |  | 1.00 (0.99-1.00, p=0.94) | 1.00 (0.99-1.01, p=0.92) |
| Diastolic blood pressure, per mmHg increase |  |  | 0.99 (0.99-1.00, p=0.15) | 1.00 (0.99-1.01, p=0.74) |
| Haemoglobin, per g/L increase |  |  | 1.00 (0.99-1.00, p=0.20) | 0.99 (0.98-0.99, p=0.01) |
| Albumin, per g/L increase |  |  | 0.96 (0.94-0.98, p=0.001) | 0.98 (0.95-1.01, p=0.16) |
| ACEi/ARB | No | 51.0 (45.4-57.3) | Reference | |
|  | Yes | 44.1 (31.9-60.8) | 0.84 (0.60-1.19, p=0.32) | 0.98 (0.60-1.60, p=0.94) |
| Triple (standard) immunosuppression regimen | No | 63.8 (54.5-74.6) | Reference | |
|  | Yes | 41.6 (35.7-48.5) | 0.66 (0.53-0.82, p<0.001) | 0.72 (0.55-0.94, p=0.02) |
| ACEi, angiotensin-converting enzyme inhibitor; ARB, angiotensin receptor blocker; AVF, arteriovenous fistula; GN, glomerulonephritis; KT, kidney transplantation | | | | |

**Table S4.** Factors associated with death-censored kidney transplant failure after KT following risk-adjustment

|  | | Incidence rates, person-years (95% CI) | Univariable HR (95% CI) | Multivariable HR (95% CI) |
| --- | --- | --- | --- | --- |
| AVF | No | 23.1 (18.2-29.3) | Reference | |
|  | Yes | 27.4 (22.4-33.5) | 1.19 (0.87-1.62, p=0.28) | 1.22 (0.76-1.95, p=0.41) |
| Duration of dialysis, per year increase |  |  | 1.01 (0.99-1.03, p=0.43) | 1.00 (0.97-1.02, p=0.78) |
| Sex | Male | 23.7 (19.3-29.1) | Reference | |
|  | Female | 28.1 (22.3-35.5) | 1.18 (0.86-1.60, p=0.30) | 1.21 (0.82-1.80, p=0.34) |
| Age, per year increase |  |  | 0.99 (0.98-1.00, p=0.03) | 0.99 (0.98-1.01, p=0.27) |
| Renal diagnosis, category | GN | 23.3 (17.7-30.6) | Reference | |
|  | Congenital | 24.6 (18.6-32.6) | 1.07 (0.72-1.58, p=0.75) | 0.93 (0.56-1.55, p=0.79) |
|  | Vascular | 22.0 (14.0-34.4) | 0.91 (0.53-1.54, p=0.71) | 0.92 (0.43-1.98, p=0.83) |
|  | Other | 32.3 (24.1-43.5) | 1.39 (0.93-2.08, p=0.11) | 1.22 (0.75-1.97, p=0.43) |
| Diabetes | No | 26.0 (22.1-30.5) | Reference | |
|  | Yes | 21.5 (13.2-35.1) | 0.78 (0.47-1.31, p=0.35) | 0.91 (0.42-1.96, p=0.81) |
| Myocardial infarction | No | 26.1 (22.3-30.5) | Reference | |
|  | Yes | 17.3 (8.7-34.6) | 0.66 (0.33-1.35, p=0.26) | 0.44 (0.16-1.23, p=0.12) |
| Atrial fibrillation | No | 25.8 (22.1-30.2) | Reference | |
|  | Yes | 14.2 (4.6-44.1) | 0.53 (0.17-1.67, p=0.28) | 0.80 (0.25-2.58, p=0.71) |
| Stroke | No | 25.0 (21.4-29.3) | Reference | |
|  | Yes | 37.4 (18.7-74.8) | 1.43 (0.70-2.92, p=0.32) | 1.21 (0.51-2.83, p=0.67) |
| Peripheral arterial disease | No | 25.3 (21.7-29.6) | Reference | |
|  | Yes | 32.6 (12.2-86.8) | 1.21 (0.45-3.28, p=0.70) | 2.02 (0.67-6.08, p=0.21) |
| Systolic blood pressure, per mmHg increase |  |  | 1.00 (0.99-1.01, p=0.69) | 1.00 (0.99-1.01, p=0.82) |
| Diastolic blood pressure, per mmHg increase |  |  | 1.00 (0.99-1.01, p=0.86) | 1.00 (0.99-1.02, p=0.92) |
| Haemoglobin, per g/L increase |  |  | 1.00 (0,99-1.00, p=0.74) | 1.00 (0.99-1.00, p=0.51) |
| Albumin, per g/L increase |  |  | 1.00 (0.97-1.04, p=0.92) | 1.00 (0.95-1.05, p=0.97) |
| ACEi/ARB | No | 25.7 (21.8-30.3) | Reference | |
|  | Yes | 23.8 (15.4-36.9) | 0.88 (0.55-1.41, p=0.60) | 1.15 (0.61-2.17, p=0.67) |
| Triple (standard) immunosuppression regimen | No | 37.0 (30.1-45.4) | Reference | |
|  | Yes | 18.3 (14.5-23.0) | 0.49 (0.36-0.67, p<0.001) | 0.60 (0.41-0.88, p=0.01) |
| ACEi, angiotensin-converting enzyme inhibitor; ARB, angiotensin receptor blocker; AVF, arteriovenous fistula; GN, glomerulonephritis; KT, kidney transplantation | | | | |

**Table S5.** Factors associated with *de novo* heart failure with preserved ejection fraction (HFpEF) after KT following risk-adjustment

|  | | Incidence rates, person-years (95% CI) | Univariable HR (95% CI) | Multivariable HR (95% CI) | Competing risks HR (95% CI) |
| --- | --- | --- | --- | --- | --- |
| AVF | No | 25.3 (20.0-32.0) | Reference | | |
|  | Yes | 49.0 (41.7-57.6) | 1.94 (1.46-2.58, p<0.001) | 2.13 (1.34-3.38, p=0.001) | 1.94 (1.25-3.03, p=0.003) |
| Duration of dialysis, per year increase |  |  | 1.03 (1.01-1.04, p<0.001) | 1.02 (1.01-1.04, p=0.001) | 1.02 (1.01-1.04, p<0.001) |
| Sex | Male | 32.0 (26.6-38.5) | Reference | | |
|  | Female | 46.6 (38.5-56.4) | 1.46 (1.12-1.90, p=0.006) | 1.83 (1.30-2.57, p=0.001) | 1.70 (1.21-2.39, p=0.002) |
| Age, per year increase |  |  | 1.03 (1.02-1.05, p<0.001) | 1.03 (1.02-1.05, p<0.001) | 1.03 (1.01-1.04, p<0.001) |
| Renal diagnosis, category | GN | 36.8 (29.3-46.3) | Reference | | |
|  | Congenital | 35.9 (28.1-45.9) | 0.96 (0.69-1.34, p=0.82) | 0.83 (0.54-1.26, p=0.39) | 0.87 (0.57-1.33, p=0.51) |
|  | Vascular | 40.6 (28.7-57.5) | 1.10 (0.72-1.67, p=0.66) | 0.64 (0.34-1.19, p=0.16) | 0.66 (0.34-1.30, p=0.23) |
|  | Other | 39.8 (30.1-52.6) | 1.04 (0.72-1.50, p=0.83) | 0.96 (0.62-1.47, p=0.83) | 0.93 (0.61-1.43, p=0.74) |
| Diabetes | No | 36.1 (31.3-41.7) | Reference | | |
|  | Yes | 50.2 (35.7-70.6) | 1.45 (0.99-2.10, p=0.06) | 1.85 (1.03-3.31, p=0.04) | 1.59 (0.82-3.06, p=0.17) |
| Myocardial infarction | No | 35.5 (30.8-40.9) | Reference | | |
|  | Yes | 68.5 (46.6-100.6) | 1.89 (1.25-2.84, p=0.002) | 1.14 (0.68-1.91, p=0.63) | 1.21 (0.72-2.04, p=0.47) |
| Atrial fibrillation | No | 36.0 (31.4-41.3) | Reference | | |
|  | Yes | 93.5 (57.3-152.6) | 2.59 (1.55-4.31, p<0.001) | 1.55 (0.83-2.91, p=0.17) | 1.55 (0.85-2.83, p=0.16) |
| Stroke | No | 36.7 (32.0-42.1) | Reference | | |
|  | Yes | 65.6 (38.1-112.9) | 1.80 (1.03-3.15, p=0.04) | 0.62 (0.26-1.45, p=0.27) | 0.64 (0.29-1.42, p=0.27) |
| Peripheral arterial disease | No | 37.8 (33.0-43.2) | Reference | | |
|  | Yes | 34.8 (13.0-92.6) | 0.96 (0.36-2.58, p=0.94) | 0.54 (0.16-1.81, p=0.32) | 0.55 (0.16-1.89, p=0.34) |
| Systolic blood pressure, per mmHg increase |  |  | 1.00 (1.00-1.01, p=0.48) | 1.00 (1.00-1.01, p=0.23) | 1.00 (1.00-1.01, p=0.23) |
| Diastolic blood pressure, per mmHg increase |  |  | 0.99 (0.98-1.00, p=0.07) | 0.99 (0.98-1.01, p=0.31) | 0.99 (0.98-1.01, p=0.40) |
| Haemoglobin, per g/L increase |  |  | 1.00 (1.00-1.01, p=0.32) | 1.00 (1.00-1.01, p=0.93) | 1.00 (1.00-1.01, p=0.68) |
| Albumin, per g/L increase |  |  | 0.97 (0.94-0.99, p=0.04) | 0.99 (0.95-1.03, p=0.71) | 1.00 (0.96-1.05, p=0.86) |
| ACEi/ARB | No | 38.8 (33.8-44.7) | Reference | | |
|  | Yes | 30.1 (20.0-45.3) | 0.78 (0.51-1.21, p=0.27) | 1.08 (0.61-1.88, p=0.80) | 1.04 (0.59-1.81, p=0.90) |
| Triple (standard) immunosuppression regimen | No | 37.7 (30.5-46.7) | Reference | | |
|  | Yes | 37.7 (31.8-44.6) | 1.05 (0.80-1.38, p=0.75) | 1.05 (0.76-1.47, p=0.75) | 1.14 (0.83-1.58, p=0.42) |
| ACEi, angiotensin-converting enzyme inhibitor; ARB, angiotensin receptor blocker; AVF, arteriovenous fistula; CV, cardiovascular; GN, glomerulonephritis; KT, kidney transplantation | | | | | |

**Table S6.** Factors associated with *de novo* heart failure with reduced ejection fraction (HFrEF) after KT following risk-adjustment

|  | | Incidence rates, person-years (95% CI) | Univariable HR (95% CI) | Multivariable HR (95% CI) | Competing risks HR (95% CI) |
| --- | --- | --- | --- | --- | --- |
| AVF | No | 12.3 (8.8-17.2) | Reference | | |
|  | Yes | 20.2 (15.7-26.0) | 1.56 (1.02-2.37, p=0.04) | 2.21 (1.10-4.46, p=0.03) | 2.02 (1.03-3.97, p=0.04) |
| Duration of dialysis, per year increase |  |  | 1.03 (1.02-1.04, p<0.001) | 1.04 (1.02-1.06, p<0.001) | 1.04 (1.02-1.05, p<0.001) |
| Sex | Male | 15.3 (11.7-20.0) | Reference | | |
|  | Female | 18.2 (13.4-24.7) | 1.17 (0.78-1.75, p=0.46) | 1.72 (0.99-2.96, p=0.07) | 1.61 (0.94-2.75, p=0.08) |
| Age, per year increase |  |  | 1.05 (1.02-1.08, p<0.001) | 1.04 (1.01-1.08, p=0.05) | 1.04 (1.02-1.07, p<0.001) |
| Renal diagnosis, category | GN | 12.6 (8.5-18.7) | Reference | | |
|  | Congenital | 14.6 (9.9-21.4) | 1.15 (0.66-1.99, p=0.63) | 1.46 (0.72-2.98, p=0.30) | 1.44 (0.71-2.89, p=0.31) |
|  | Vascular | 24.1 (15.4-37.8) | 1.91 (1.05-3.47, p=0.03) | 0.79 (0.30-2.06, p=0.63) | 0.75 (0.31-1.78, p=0.51) |
|  | Other | 20.3 (13.7-30.0) | 1.61 (0.92-2.80, p=0.09) | 1.60 (0.76-3.35, p=0.21) | 1.42 (0.69-2.92, p=0.35) |
| Diabetes | No | 13.3 (10.5-16.8) | Reference | | |
|  | Yes | 41.0 (28.2-59.9) | 3.11 (1.98-4.88, p<0.001) | 4.98 (2.37-10.46, p<0.001) | 3.86 (2.00-7.43, p<0.001) |
| Myocardial infarction | No | 14.4 (11.6-18.0) | Reference | | |
|  | Yes | 44.8 (27.8-72.1) | 2.93 (1.73-4.95, p<0.001) | 2.32 (1.22-4.42, p=0.01) | 2.40 (1.27-4.53, p=0.007) |
| Atrial fibrillation | No | 16.0 (13.0-19.7) | Reference | | |
|  | Yes | 29.2 (12.2-70.2) | 1.35 (0.33-5.58, p=0.68) | 0.53 (0.07-4.39, p=0.56) | 0.75 (0.20-2.77, p=0.66) |
| Stroke | No | 16.1 (13.1-19.8) | Reference | | |
|  | Yes | 25.2 (10.5-60.6) | 1.60 (0.65-3.95, p=0.31) | 0.69 (0.16-2.90, p=0.61) | 0.55 (0.14-2.13, p=0.39) |
| Peripheral arterial disease | No | 16.1 (13.1-19.7) | Reference | | |
|  | Yes | 34.8 (13.0-92.6) | 2.28 (0.83-6.21, p=0.11) | 0.95 (0.26-3.46, p=0.94) | 1.00 (0.26-3.79, p=0.99) |
| Systolic blood pressure, per mmHg increase |  |  | 1.00 (0.99-1.02, p=0.88) | 1.00 (0.98-1.02, p=0.96) | 1.00 (0.99-1.01. p=0.60) |
| Diastolic blood pressure, per mmHg increase |  |  | 0.98 (0.96-1.01, p=0.15) | 1.00 (0.97-1.02, p=0.76) | 1.00 (0.98-1.01, p=0.70) |
| Haemoglobin, per g/L increase |  |  | 0.99 (0.98-0.99, p=0.02) | 0.99 (0.98-1.00, p=0.18) | 1.00 (0.99-1.01, p=0.83) |
| Albumin, per g/L increase |  |  | 0.97 (0.91-1.03, p=0.34) | 1.00 (0.91-1.09, p=0.92) | 0.97 (0.92-1.03, p=0.32) |
| ACEi/ARB | No | 15.7 (12.6-19.6) | Reference | | |
|  | Yes | 21.0 (12.8-34.2) | 2.05 (1.06-3.96 (p=0.03) | 3.67 (1.41-9.4, p=0.008) | 1.84 (0.88-3.86, p=0.10) |
| Triple (standard) immunosuppression regimen | No | 19.8 (14.7-26.6) | Reference | | |
|  | Yes | 14.3 (10.9-18.9) | 0.75 (0.42-1.32, p=0.31) | 0.42 (0.19-0.93, p=0.03) | 0.74 (0.45-1.23, p=0.24) |
| ACEi, angiotensin-converting enzyme inhibitor; ARB, angiotensin receptor blocker; AVF, arteriovenous fistula; CV, cardiovascular; GN, glomerulonephritis; KT, kidney transplantation | | | | | |

**Table S7.** Factors associated with de novo heart failure after KT following risk-adjustment (sensitivity analysis including only haemodialysis patients pre-transplantation - AVF vs. CVC, n=923)

|  | | Incidence rates, person-years (95% CI) | Univariable HR (95% CI) | Multivariable HR (95% CI) |
| --- | --- | --- | --- | --- |
| HD access type | CVC | 33.8 (23.9-47.8) | Reference | |
|  | AVF | 57.9 (50.0-67.2) | 1.70 (1.16-2.47, p=0.006) | 2.13 (1.40-3.25, p<0.001) |
| Duration of dialysis, per year increase |  |  | 1.03 (1.01-1.04, p<0.001) | 1.03 (1.01-1.04, p<0.001) |
| Sex | Male | 39.7 (33.6-46.8) | Reference | |
|  | Female | 55.9 (47.0-66.6) | 1.41 (1.11-1.79, p=0.005) | 1.92 (1.40-2.64, p<0.001) |
| Age, per year increase |  |  | 1.04 (1.03-1.05, p<0.001) | 1.03 (1.02-1.05, p<0.001) |
| Renal diagnosis, category | GN | 42.4 (34.2-52.5) | Reference | |
|  | Congenital | 43.2 (34.6-54.0) | 1.01 (0.74-1.37, p=0.96) | 0.87 (0.59-1.29, p=0.48) |
|  | Vascular | 55.9 (41.6-75.1) | 1.30 (0.90-1.88, p=0.16) | 0.60 (0.34-1.06, p=0.08) |
|  | Other | 49.5 (38.5-63.6) | 1.13 (0.81-1.58, p=0.46) | 1.02 (0.69-1.52, p=0.92) |
| Diabetes | No | 42.3 (37.1-48.4) | Reference | |
|  | Yes | 74.6 (56.4-98.7) | 1.81 (1.32-2.47, p<0.001) | 2.42 (1.47-3.99, p=0.001) |
| Myocardial infarction | No | 42.9 (37.8-48.8) | Reference | |
|  | Yes | 89.6 (64.0-125.4) | 2.04 (1.42-2.92, p<0.001) | 1.33 (0.85-2.08, p=0.21) |
| Atrial fibrillation | No | 44.2 (39.0-50.1) | Reference | |
|  | Yes | 105.2 (66.3-166.9) | 2.35 (1.46-3.79, p<0.001) | 1.36 (0.75-2.47, p=0.31) |
| Stroke | No | 44.6 (39.4-50.5) | Reference | |
|  | Yes | 85.7 (53.3-137.9) | 1.92 (1.17-3.14, p=0.009) | 0.57 (0.26-1.25, p=0.16) |
| Peripheral arterial disease | No | 45.9 (40.6-51.8) | Reference | |
|  | Yes | 52.1 (23.4-116.0) | 1.17 (0.52-2.62, p=0.71) | 0.71 (0.27-1.83, p=0.48) |
| Systolic blood pressure, per mmHg increase |  |  | 1.00 (0.99-1.01, p=0.48) | 1.00 (1.00-1.01, 0.31) |
| Diastolic blood pressure, per mmHg increase |  |  | 0.99 (0.98-0.99, p=0.03) | 0.99 (0.98-1.01, 0.35) |
| Haemoglobin, per g/L increase |  |  | 1.00 (0.99-1.00, p=0.94) | 1.00 (0.99-1.00, p=0.54) |
| Albumin, per g/L increase |  |  | 0.97 (0.95-0.99, p=0.02) | 0.99 (0.96-1.03, p=0.70) |
| ACEi/ARB | No | 46.0 (40.5 (52.4) | Reference | |
|  | Yes | 45.9 (32.9-63.9) | 0.99 (0.69-1.42, p=0.97) | 1.41 (0.89-2.25, p=0.15) |
| Triple (standard) immunosuppression regimen | No | 47.6 (39.4-57.6) | Reference | |
|  | Yes | 45.0 (38.5-52.5) | 0.98 (0.77-1.26, p=0.89) | 0.93 (0.69-1.26, p=0.65) |
| ACEi, angiotensin-converting enzyme inhibitor; ARB, angiotensin receptor blocker; AVF, arteriovenous fistula; CVC, central venous catheter; GN, glomerulonephritis; HD, haemodialysis; KT, kidney transplantation | | | | |

**Table S8.** Factors associated with de novo heart failure after KT following risk-adjustment (sensitivity analysis per AVF type, n=716)

|  | | Incidence rates, person-years (95% CI) | Univariable HR (95% CI) | Multivariable HR (95% CI) |
| --- | --- | --- | --- | --- |
| AVF type | RCF | 44.7 (32.8-60.9) | Reference | |
|  | BCF | 64.2 (47.4-86.9) | 1.43 (0.93-2.21, p=0.10) | 1.45 (0.88-2.38, p=0.14) |
|  | BBF | 53.4 (31.6-90.1) | 1.26 (0.69-2.33, p=0.45) | 1.20 (0.62-2.32, p=0.58) |
|  | Other/Unknown | 65.4 (52.4-81.5) | 1.48 (1.01-2.16, p=0.05) | 1.55 (0.99-2.43, p=0.06) |
| Duration of dialysis, per year increase |  |  | 1.03 (1.01-1.04, p<0.001) | 1.02 (1.01-1.04, p=0.001) |
| Sex | Male | 39.7 (33.6-46.8) | Reference | |
|  | Female | 55.9 (47.0-66.6) | 1.41 (1.11-1.79, p=0.005) | 1.69 (1.18-2.43, p=0.004) |
| Age, per year increase |  |  | 1.04 (1.03-1.05, p<0.001) | 1.03 (1.02-1.05, p<0.001) |
| Renal diagnosis, category | GN | 42.4 (34.2-52.5) | Reference | |
|  | Congenital | 43.2 (34.6-54.0) | 1.01 (0.74-1.37, p=0.96) | 0.84 (0.54-1.29, p=0.42) |
|  | Vascular | 55.9 (41.6-75.1) | 1.30 (0.90-1.88, p=0.16) | 0.65 (0.36-1.19, p=0.17) |
|  | Other | 49.5 (38.5-63.6) | 1.13 (0.81-1.58, p=0.46) | 0.95 (0.61-1.48, p=0.84) |
| Diabetes | No | 42.3 (37.1-48.4) | Reference | |
|  | Yes | 74.6 (56.4-98.7) | 1.81 (1.32-2.47, p<0.001) | 2.23 (1.28-3.87, p=0.005) |
| Myocardial infarction | No | 42.9 (37.8-48.8) | Reference | |
|  | Yes | 89.6 (64.0-125.4) | 2.04 (1.42-2.92, p<0.001) | 1.48 (0.89-2.46, p=0.13) |
| Atrial fibrillation | No | 44.2 (39.0-50.1) | Reference | |
|  | Yes | 105.2 (66.3-166.9) | 2.35 (1.46-3.79, p<0.001) | 1.36 (.072-2.55, p=0.34) |
| Stroke | No | 44.6 (39.4-50.5) | Reference | |
|  | Yes | 85.7 (53.3-137.9) | 1.92 (1.17-3.14, p=0.009) | 0.40 (0.16-1.04, p=0.06) |
| Peripheral arterial disease | No | 45.9 (40.6-51.8) | Reference | |
|  | Yes | 52.1 (23.4-116.0) | 1.17 (0.52-2.62, p=0.71) | 0.64 (0.22-1.84, p=0.40) |
| Systolic blood pressure, per mmHg increase |  |  | 1.00 (1.00-1.01, p=0.48) | 1.00 (1.00-1.01, p=0.30) |
| Diastolic blood pressure, per mmHg increase |  |  | 0.99 (0.98-0.99, p=0.03) | 0.99 (0.98-1.01, p=0.31) |
| Haemoglobin, per g/L increase |  |  | 1.00 (0.99-1.00, p=0.94) | 1.00 (0.99-1.00, p=0.24) |
| Albumin, per g/L increase |  |  | 0.97 (0.95-0.99, p=0.02) | 0.99 (0.95-1.03, p=0.59) |
| ACEi/ARB | No | 46.0 (40.5-52.4) | Reference | |
|  | Yes | 45.9 (32.9-63.9) | 0.99 (0.69-1.42, p=0.97) | 1.16 (0.69-1.95, p=0.59) |
| Triple (standard) immunosuppression regimen | No | 47.6 (39.4-57.6) | Reference | |
|  | Yes | 45.0 (38.5-52.5) | 0.98 (0.77-1.26, p=0.89) | 0.91 (0.65-1.26, p=0.56) |
| ACEi, angiotensin-converting enzyme inhibitor; ARB, angiotensin receptor blocker; AVF, arteriovenous fistula; BBF, brachiobasilic fistula; BCF, brachiocephalic fistula; GN, glomerulonephritis; KT, kidney transplantation; RCF, radiocephalic fistula | | | | |

**STROBE statement**. Checklist of items that should be included in observational studies using routinely collected health data

|  | Item No | Recommendation | Location in manuscript where items are reported |
| --- | --- | --- | --- |
| **Title and abstract** | 1 | (*a*) Indicate the study’s design with a commonly used term in the title or the abstract | (*a*) Abstract |
|  |  | (*b*) Provide in the abstract an informative and balanced summary of what was done and what was found | (*b*) Abstract |
| Introduction | | |  |
| Background/rationale | 2 | Explain the scientific background and rationale for the investigation being reported | Introduction (paragraphs 1 and 2) |
| Objectives | 3 | State specific objectives, including any prespecified hypotheses | Introduction (paragraph 3) |
| Methods | | |  |
| Study design | 4 | Present key elements of study design early in the paper | Materials and methods (study variables, outcome definitions and patient allocation) |
| Setting | 5 | Describe the setting, locations, and relevant dates, including periods of recruitment, exposure, follow-up, and data collection | Materials and methods (data sources and study population) |
| Participants | 6 | (*a*) Give the eligibility criteria, and the sources and methods of selection of participants. Describe methods of follow-up | (*a*) Materials and methods (data sources and study population) |
|  |  | (*b*) For matched studies, give matching criteria and number of exposed and unexposed | N/a |
| Variables | 7 | Clearly define all outcomes, exposures, predictors, potential confounders, and effect modifiers. Give diagnostic criteria, if applicable | Materials and methods (study variables, outcome definitions, patient allocation and table 1) |
| Data sources/ measurement | 8 | For each variable of interest, give sources of data and details of methods of assessment (measurement). Describe comparability of assessment methods if there is more than one group | Materials and methods (study variables, outcome definitions and patient allocation) |
| Bias | 9 | Describe any efforts to address potential sources of bias | Materials and methods (statistical analyses, propensity score matching - paragraph 4) |
| Study size | 10 | Explain how the study size was arrived at | Materials and methods (all) |
| Quantitative variables | 11 | Explain how quantitative variables were handled in the analyses. If applicable, describe which groupings were chosen and why | Materials and methods (study variables, outcome definitions and statistical analyses) |
| Statistical methods | 12 | (*a*) Describe all statistical methods, including those used to control for confounding | (*a*) Materials and methods (study variables, statistical analyses, propensity score matching, ROC curves – all paragraphs) |
|  |  | (*b*) Describe any methods used to examine subgroups and interactions | (*b*) Materials and methods (study variables, statistical analyses, propensity score matching) |
|  |  | (*c*) Explain how missing data were addressed | *(c*) Materials and methods (statistical analyses) |
|  |  | (*d*) If applicable, explain how loss to follow-up was addressed | (*d*) Materials and methods (statistical analyses, censoring) |
|  |  | (*e*) Describe any sensitivity analyses | (*e*) Materials and methods (sensitivity analyses, paragraphs 3, 4 and 5) |
| Results | | |  |
| Participants | 13* | (a) Report numbers of individuals at each stage of study—eg numbers potentially eligible, examined for eligibility, confirmed eligible, included in the study, completing follow-up, and analysed | (a) Results (baseline patient characteristics, figure 1, table 2) |
|  |  | (b) Give reasons for non-participation at each stage | (b) Results (baseline patient characteristics, figure 1) |
|  |  | (c) Consider use of a flow diagram | (c) Figure 1 |
| Descriptive data | 14 | (a) Give characteristics of study participants (eg demographic, clinical, social) and information on exposures and potential confounders | (a) Results (baseline patient characteristics, table 2) |
|  |  | (b) Indicate number of participants with missing data for each variable of interest | (b) Methods (statistical analyses, paragraph 3) |
|  |  | (c) Summarise follow-up time (eg, average and total amount) | (c) Results (incidence of de novo HF after transplantation, paragraph 2) |
| Outcome data | 15 | Report numbers of outcome events or summary measures over time | Results (incidence of de novo HF after transplantation, table 3) |
| Main results | 16 | (*a*) Give unadjusted estimates and, if applicable, confounder-adjusted estimates and their precision (eg, 95% confidence interval). Make clear which confounders were adjusted for and why they were included | (*a*) Results (incidence of de novo HF after transplantation, independent predictors of de novo heart failure, table 4 and 5) |
|  |  | (*b*) Report category boundaries when continuous variables were categorized | (*a*) Results (all) |
|  |  | (*c*) If relevant, consider translating estimates of relative risk into absolute risk for a meaningful time period | (*c*) Not relevant |
| Other analyses | 17 | Report other analyses done—eg analyses of subgroups and interactions, and sensitivity analyses | Results (sensitivity analyses) |
| Discussion | | |  |
| Key results | 18 | Summarise key results with reference to study objectives | Discussion (paragraph 1) |
| Limitations | 19 | Discuss limitations of the study, taking into account sources of potential bias or imprecision. Discuss both direction and magnitude of any potential bias | Discussion (paragraph 8) |
| Interpretation | 20 | Give a cautious overall interpretation of results considering objectives, limitations, multiplicity of analyses, results from similar studies, and other relevant evidence | Discussion (throughout) |
| Generalisability | 21 | Discuss the generalisability (external validity) of the study results | Discussion (paragraph 9) |
| Other information | | |  |
| Funding | 22 | Give the source of funding and the role of the funders for the present study and, if applicable, for the original study on which the present article is based | Funding statement |
